# Sleep Quality, Dietary Patterns, and Nutrition Knowledge in Ultramarathon Runners and American Football Players: A Comparative Cross-Sectional Study

**DOI:** 10.3390/nu18091322

**Published:** 2026-04-22

**Authors:** Aureliusz Andrzej Kosendiak, Bartosz Colinso, Zofia Kuźnik, Szymon Makles, Hanna Bazan, Weronika Hariasz, Elżbieta Biernat

**Affiliations:** 1 College of Health Studies, University of Lower Silesia, 53-611 Wroclaw, Poland; 2 Medical Faculty, Wroclaw Medical University, 50-345 Wroclaw, Poland; 3 Medical Faculty, University of Opole, 45-040 Opole, Poland; 4 Tourism Economy Research Unit, Institute of International Economic Policy, Collegium of World Economy, SGH Warsaw School of Economics, 02-554 Warsaw, Poland

**Keywords:** sleep quality, nutrition knowledge, sports nutrition, American football, ultramarathon

## Abstract

**Background**: Nutrition and sleep are critical determinants of athletic performance and recovery. Direct comparative research between endurance and strength–power athletes remains limited. This study aimed to evaluate and compare nutritional knowledge, dietary habits, sleep quality, and Body Mass Index between ultramarathon runners and American football players, as well as to explore independent predictors of sleep quality. **Methods**: A cross-sectional study was conducted among 231 male athletes. To address group size disparity and mitigate statistical bias, a random undersampling technique was applied to create a balanced cohort of 86 athletes comprising 43 ultramarathon runners and 43 American football players. Nutritional parameters were assessed using the Kom-PAN questionnaire. Sleep quality was evaluated using the Pittsburgh Sleep Quality Index. Between-group comparisons were performed using the Mann–Whitney U test with False Discovery Rate correction. An integrated multiple regression model was constructed to identify predictors of global sleep quality. **Results**: Ultramarathon runners demonstrated significantly better overall sleep quality (*p* = 0.026) and higher nutritional knowledge (*p* < 0.001) compared to American football players. Differences in adherence to pro-healthy and non-healthy dietary patterns were not statistically significant after False Discovery Rate correction. The integrated multiple regression model revealed that the athletic discipline was the primary independent predictor of global sleep quality (*p* = 0.001), while dietary variables did not exhibit a significant independent effect. Furthermore, higher Body Mass Index was independently associated with better sleep scores within the multivariate model (*p* = 0.008). **Conclusions**: Significant sport-specific differences exist in BMI, nutritional knowledge, and sleep quality. Global sleep quality appears to be primarily associated with the specific physiological and environmental demands of the athletic discipline rather than individual dietary factors, which were not independently significant in the multivariable model. These findings suggest that recovery strategies in strength–power athletes may require a broader, multifactorial approach beyond nutritional education alone.

## 1. Introduction

Nutrition and sleep quality represent two closely linked determinants of athletic performance, recovery capacity, and long-term physiological health. Adequate nutritional intake provides the substrate for energy production, promotes muscle protein synthesis, restores glycogen reserves, and modulates metabolic and endocrine processes that support sustained physical output [1]. Sleep, in parallel, is a fundamental biological function essential for cognitive performance, neuromuscular coordination, immune regulation, and recovery from training-induced stress [2,3]. In athletic populations, the interaction between these factors is particularly critical. Inappropriate dietary patterns may disrupt sleep through altered hormonal signaling, glycemic instability, or gastrointestinal distress [4,5,6], whereas insufficient or fragmented sleep can impair appetite control, metabolic efficiency, and nutrient assimilation [7,8]. This reciprocal relationship underscores the need for integrated strategies that address both nutrition and sleep as components of comprehensive athlete care.

This investigation centers on two markedly different athletic groups: ultramarathon runners (UMs) and American football players (AFs), selected because of their contrasting physiological profiles and training demands. UMs are endurance athletes who perform prolonged bouts of predominantly aerobic exercise, often exceeding 50–100 km in competition [9]. Their performance relies heavily on oxidative metabolism and is supported by adaptations such as low body fat levels [10], elevated mitochondrial content, extensive capillary development, and superior cardiovascular efficiency [11]. From a nutritional perspective, these athletes require approaches that maximize glycogen availability, preserve electrolyte balance, and facilitate recovery from sustained metabolic and muscular load. Such strategies commonly emphasize carbohydrate availability, moderate protein intake, and diets rich in micronutrients [12].

Conversely, AFs engage in intermittent, high-intensity activity characterized by short-duration explosive efforts, rapid directional changes, and repeated maximal or near-maximal strength outputs [13,14]. Their physical characteristics differ substantially, with greater muscle mass, larger cross-sectional muscle area, and body compositions optimized for power production and physical contact [15]. The capacity to rapidly produce high levels of force and power via the phosphagen and glycolytic energy systems is essential for actions such as sprinting, tackling, blocking, and other explosive, position-specific movements. Consequently, well-developed anaerobic capacity and metabolic efficiency represent key physiological determinants of performance in American football [16]. Nutritional priorities in this population include sufficient protein intake to support muscle hypertrophy, carefully timed carbohydrates to fuel intense activity, and high overall energy consumption to meet caloric requirements [17,18]. Sleep plays a crucial role in this group, as the combination of intense training and mechanical stress from collisions necessitates substantial recovery to reduce injury risk, enhance strength adaptations, and preserve cognitive function for tactical performance [19,20].

The pronounced differences between these two athlete populations, endurance-based versus power-based, aerobic versus anaerobic metabolism, and lean versus muscular body composition, create a valuable framework for examining sport-specific demands and performing an exploratory comparison of baseline lifestyle behaviors such as dietary practices, nutrition-related knowledge, and sleep quality. Although considerable research has explored nutrition and sleep independently within specific sports, direct comparisons between athlete groups with such divergent metabolic and physical characteristics remain limited.

The influence of specific sport disciplines on athlete physiology is multidimensional. These differences extend beyond metabolic adaptations and body compositions. They also encompass other biological parameters determined by the training environment. Research indicates that the type of sport practiced significantly modifies oral health status and salivary parameters in athletes [21]. The lack of comparative research on sleep quality and nutritional habits between predominantly aerobic and anaerobic disciplines underscores the necessity of formulating discipline-specific recommendations that are precisely aligned with the underlying physiological demands, including, for example, the identification of optimal sleep patterns to support recovery and performance, as well as the development of tailored dietary strategies that best meet the metabolic and nutritional requirements characteristic of a given discipline. This gap is significant, as extrapolating findings from one sport to another may result in recommendations that are not optimally aligned with sport-specific needs. Therefore, the present study, by describing and comparing these parameters between these two disciplines, may serve as an introduction to the development of such recommendations.

Accordingly, the main objective of this study was to compare nutritional knowledge, dietary behaviors, and sleep quality, and Body Mass Index (BMI) between UM and AF. By analyzing two groups situated at opposite ends of the spectrum in terms of energy requirements, training intensity, and physiological phenotype, this research aims to document these differences and provide the empirical data necessary for the development of discipline-specific recommendations. They may contribute to the development of targeted interventions that enhance performance and support long-term health, reinforcing the importance of individualized approaches in sports nutrition and sleep management.

## 2. Materials and Methods

### 2.1. Study Design and Participants

This cross-sectional study was conducted between January and March 2021. Its primary aim was to compare dietary patterns, nutrition knowledge, and sleep quality between UM and AF. The study group consisted of active professional players of the Panthers Wroclaw American football team (*n* = 43), while the comparison group included ultra-endurance runners (*n* = 188).

The study protocol was approved by the Bioethics Committee of Wroclaw Medical University (approval no. KB-22/2021 and KB-601/2021). All participants provided written informed consent prior to participation. The study was conducted in accordance with the Declaration of Helsinki. Participation was voluntary and anonymous.

**Figure 1:**
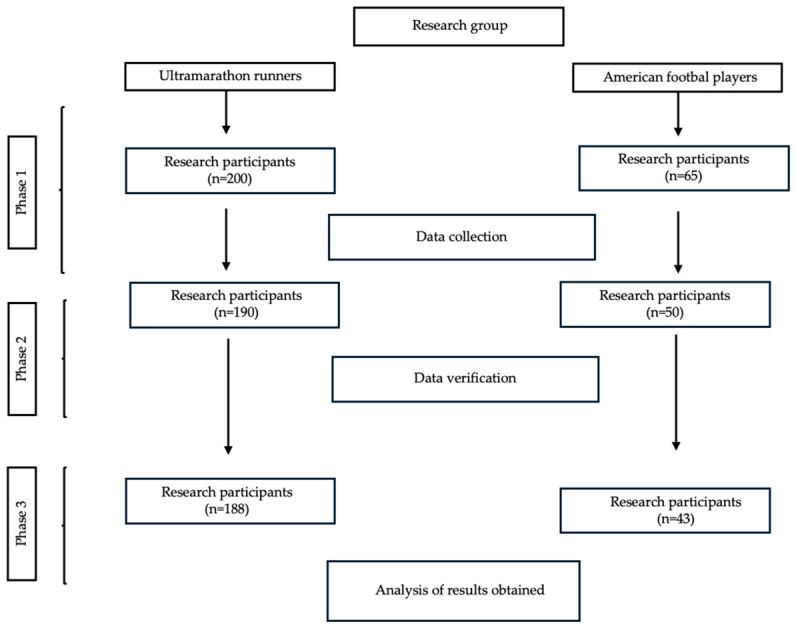
Study selection process.

Participants were recruited from active club athletes competing in either endurance (ultramarathon) or strength–power (American football) disciplines. To be eligible for inclusion, athletes had to be active club members, aged 18 to 30 years, male, and free from chronic illnesses and provide written consent to participate in the study. Athletes were excluded if they did not meet these criteria, including being under 18 years of age, having any chronic illness, lacking club player status, or declining participation. Additional exclusions were applied after data verification: incomplete questionnaire responses or failure to comply with embedded verification questions resulted in removal from the final dataset. Only male athletes were ultimately included in the analyzed cohort. Data were collected using an electronic questionnaire that was available to participants for several weeks, with no time limit for completion. In the case of questions or concerns, participants were able to contact the project coordinator. The survey included a demographic and author-designed questionnaire, the KomPAN questionnaire for dietary habits and nutrition knowledge assessment, and the Pittsburgh Sleep Quality Index (PSQI). A detailed description of the recruitment process, along with inclusion and exclusion criteria, is presented in Figure 1.

### 2.2. KomPAN

Dietary habits and nutrition knowledge were assessed using the validated KomPAN questionnaire developed by the Polish Academy of Sciences [22]. The instrument evaluates the frequency of consumption of selected food groups and allows calculation of two indices: the Pro-Healthy Diet Index (PHD) and the Non-Healthy Diet Index (NHD), expressed as percentage values (0–100%).

Overall diet quality was determined using the Diet Quality Index (DQI), calculated as the difference between PHD and NHD scores, with possible values ranging from −100 to 100. Higher positive scores indicate a more health-promoting dietary pattern, whereas negative values reflect a predominance of unhealthy dietary components. Based on the responses, participants receive a score that reflects their dietary tendency as low (−100–−26), medium (−25–25), or high (25–100).

Nutrition knowledge was evaluated using a 25-item single-choice test included in the KomPAN questionnaire, with response options being “True,” “False,” or “I’m not sure”. Each correct answer was awarded one point, while incorrect or uncertain responses receive zero points, resulting in a total score ranging from 0 to 25. The total score reflects the participant’s level of Dietary Knowledge (DK). Based on their responses, participants receive a score that reflects their dietary tendency as insufficient (0–8), sufficient (9–16), or good (17–25).

To ensure data reliability, control questions were embedded within the survey. Responses failing validation checks were excluded from the final dataset. The KomPAN questionnaire has demonstrated satisfactory reliability and internal consistency in previous studies [23,24].

### 2.3. PSQI

Sleep quality was assessed using the PSQI [25]. This questionnaire consists of 19 self-rated items that generate seven component scores:

- Subjective sleep quality (C1): Assigning a score based on the subjective evaluation of sleep quality by the respondent.
- Sleep latency (C2): Allocating zero points for a latency period of less than 15 min and three points for a latency exceeding 60 min.
- Sleep duration (C3): Awarding zero points for achieving 7 or less hours of sleep.
- Habitual sleep efficiency (C4): Scoring contingent on the ratio of actual hours of sleep to the time spent in bed.
- Sleep disturbances (C5): A scoring system dependent on the presence of disturbances affecting the continuity of night sleep, such as feeling too hot or too cold, experiencing unsettling dreams, or discomfort in breathing.
- Use of sleep medication (C6): Scoring in relation to the frequency of sleep medication usage by the respondent.
- Daytime dysfunctions (C7): Scoring based on the frequency with which a lack of nocturnal rest impacts daytime behavioral disruptions, such as eating habits or participation in meetings.

The sum of these components yields a global score ranging from 0 to 21 points, with higher scores indicating poorer sleep quality. In the present study, a global PSQI score >5 was considered indicative of poor sleep quality. The PSQI is a validated and widely used instrument with established psychometric properties [26].

### 2.4. Statistical Analysis

Statistical analyses were performed using the R statistical environment. To address the initial disparity in group sizes and mitigate the risk of statistical bias, a random undersampling technique was employed. A subgroup of 43 participants was randomly selected from the UM cohort to match the sample size of the AF, creating a perfectly balanced dataset (*n* = 86). This approach reduced the size of the original UM sample and may have affected the representativeness of the analyzed data. A fixed random seed was utilized to ensure the full reproducibility of this procedure.

The normality of continuous variables was evaluated using the Shapiro–Wilk test. Due to the non-normal distribution of the data, non-parametric methods were applied. Categorical variables were presented as frequencies and percentages, and between-group differences were analyzed using Fisher’s exact test. Continuous variables were summarized using medians and interquartile ranges. Between-group comparisons for continuous variables were conducted using the Mann–Whitney U test. To rigorously control the False Discovery Rate (FDR) arising from multiple testing, all resulting p-values were adjusted using the Benjamini–Hochberg procedure. The magnitude of the differences was evaluated by calculating the biserial rank correlation coefficient (r) as the effect size.

To identify the independent predictors of global sleep quality and account for potential confounding factors, an integrated multiple linear regression model was constructed. The global PSQI score served as the dependent variable. The athletic group, BMI, DK, PHD, NHD scores were included as independent predictors. The assumption of no multicollinearity was verified and satisfied using the Variance Inflation Factor, yielding values below 1.6 for all variables.

To minimize the penalty arising from the False Discovery Rate (FDR) correction while preserving statistical power, correlation analyses were specifically targeted at the PSQI components that demonstrated significant between-group differences in the initial analysis (global PSQI score, sleep latency, and daytime dysfunction). This hypothesis-driven approach allowed for a reduction in the number of tested variables. Spearman’s rank correlations with FDR correction were performed independently for both athletic groups. The level of statistical significance was set at *p* < 0.05 for all analyses.

## 3. Results

### 3.1. Characteristics of the Baseline Group

The study population consisted of 188 UMs and 43 AFs, all of whom were male. Significant differences were observed in body composition and residence; while 62.8% of runners maintained a normal weight (mean BMI = 24.1), most football players (83.7%) were classified as overweight (mean BMI = 29.1). Geographical distribution showed that over half of the runners (51.1%) and nearly two-thirds of football players (65.1%) resided in cities with more than 100,000 inhabitants. Furthermore, a marked disparity in nutritional knowledge was identified, as 24.5% of runners achieved a “good” mark, whereas 100% of football players were rated as having only “sufficient” knowledge. Subjective assessments revealed that 73.4% of runners perceived their sleep as “good,” compared to only 55.8% of football players. The characteristics of the baseline group are shown in Table 1.

### 3.2. Categorical Characteristics of the Balanced Groups

To address the initial group size disparity and minimize the risk of statistical bias, an undersampling technique was employed, resulting in a perfectly balanced cohort of 86 athletes comprising 43 AF and 43 UM. The categorical characteristics of this balanced sample are presented in Table 2. A significant difference was observed in BMI categories, with the vast majority of AFs classified as overweight (83.7%), whereas most UMs maintained a normal weight (69.8%) (*p* < 0.001). DK also differed significantly between the disciplines; while all AF demonstrated sufficient knowledge, a considerable proportion of UM (27.9%) achieved a good knowledge rating (*p* < 0.001). Regarding global sleep quality categories, the AF group exhibited a higher prevalence of poor sleep (55.8%) compared to the UM group (32.6%) (*p* = 0.050). No significant categorical differences were found in the prevalence of low or medium adherence to NHD and PHD patterns.

### 3.3. Comparison of All Analyzed Continuous Variables

A detailed comparison of all analyzed continuous variables, utilizing the Mann–Whitney U test with False Discovery Rate (FDR) correction, is detailed in Table 3. The analysis confirmed robust sport-specific disparities. The largest effect size was observed for BMI, which was significantly higher in AFs than in UMs (*p* < 0.001, r = 0.632). UMs achieved significantly higher scores in continuous DK compared to AFs (*p* < 0.001, r = 0.499). Notably, after FDR correction, only the DQI score remained significantly different between groups (*p* = 0.040), while the NHD (*p* = 0.130) and PHD (*p* = 0.491) scores did not. However, global PSQI scores remained significantly higher in AFs, indicating worse overall sleep (*p* = 0.026, r = 0.280). Within the PSQI components, AFs experienced significantly worse daytime dysfunction (*p* = 0.003, r = 0.366), longer sleep latency (*p* = 0.026, r = 0.275), and poorer subjective sleep quality (*p* = 0.048, r = 0.244).

### 3.4. Integrated Multiple Regression Model for the Global Sleep Quality Score

To determine the independent predictors of global sleep quality while controlling for confounding factors, a multiple regression model was calculated, as presented in Table 4. The model incorporated the athletic group, BMI, dietary patterns, and DK as predictors of the global PSQI score. The athletic discipline emerged as a highly significant predictor, where belonging to the UM group was associated with a PSQI score reduction of 2.498 points, indicating substantially better sleep quality independent of other variables (*p* = 0.001; 95% CI: 3.976 to −1.021). Furthermore, BMI was identified as a significant predictor with a negative estimator (−0.229), suggesting that within this specific multivariate context, a higher BMI predicts a lower PSQI score (better sleep) (*p* = 0.008; 95% CI: −0.400 to −0.058). Crucially, variables related to nutrition, including DQI and DK, failed to reach statistical significance in the integrated model (all *p* > 0.05), demonstrating a lack of independent influence on sleep quality in this cohort. This association should be interpreted cautiously, as BMI may act as a surrogate marker of sport-specific phenotype rather than an independent determinant of sleep quality.

### 3.5. Targeted Spearman Correlations Between BMI and Key PSQI Components by Sport Discipline

The results of the targeted correlation analysis are presented in Table 5 and Table 6. Among AF, a higher BMI was significantly correlated with a better global PSQI score (rho = −0.350, *p* = 0.021), shorter sleep latency (rho = −0.449, *p* = 0.008), and reduced daytime dysfunction (rho = −0.392, *p* = 0.014). In contrast, no significant correlations between BMI and sleep parameters were found in the UM group (all *p* > 0.05). DQI showed no significant independent associations with key PSQI components in either group after FDR correction (all *p* > 0.05). Furthermore, a significant positive correlation between nutritional knowledge and the DQI was observed exclusively in the AF group (Table 7; rho = 0.388, *p* = 0.010).

## 4. Discussion

The present study aimed to assess and compare sleep quality, dietary patterns, nutritional knowledge, and BMI among UMs and AFs, as well as to explore the interrelationships between these variables. Our findings indicate meaningful differences between the groups, which appear to reflect the distinct physiological and performance demands of endurance versus strength–power disciplines.

### 4.1. BMI

In the analysis of BMI, most UMs were classified as normal-weight (62.8%), with 36.2% categorized as overweight. In contrast, the majority of AF were classified as overweight (83.7%). These differences likely reflect the divergent training adaptations characteristic of the two sports. BMI, although widely used, does not differentiate between fat mass and fat-free mass, including skeletal muscle and bone, which limits its validity in athletic populations [27,28]. Thus, differences in BMI between AFs and UMs should be interpreted cautiously, as they may reflect differences in lean mass rather than adiposity.

A 2023 study comparing endurance, strength, and team-sport athletes found that endurance athletes consistently exhibited the lowest values across body composition parameters, including fat mass, while strength-trained athletes demonstrated higher appendicular lean mass index (ALMI), reflecting greater relative skeletal muscle mass in the limbs [29]. Similarly, research on masters athletes showed that only strength-trained individuals maintained significantly higher ALMI compared with endurance-trained peers, whereas fat mass remained lower in sprint and endurance athletes relative to strength athletes and non-athletic controls [30]. These patterns are further supported by comparisons of Olympic weightlifters and long-distance runners, which consistently demonstrate higher BMI among strength-oriented athletes [31].

Taken together, in strength- and power-oriented disciplines such as AF, an elevated BMI likely reflects predominantly greater lean body mass, accompanied to a lesser extent by increased adipose tissue, whereas UMs exhibit lower muscle mass and reduced body fat, consistent with the metabolic and mechanical demands of endurance performance. Collectively, these patterns indicate that the higher BMI in our football cohort primarily represents increased skeletal muscle, while the lower BMI in ultramarathon runners reflects the lean, endurance-specific phenotype described in previous studies. Importantly, the observed differences in BMI between the studied groups should not be interpreted as direct differences in body fatness. In strength–power athletes such as AF, higher BMI likely reflects increased skeletal muscle mass rather than excess adiposity, which is consistent with previous findings in athletic populations [29,30,31]. Therefore, BMI in this context should be considered a proxy for sport-specific body composition rather than a precise indicator of adiposity. Accordingly, the observed associations involving BMI should be interpreted with caution.

### 4.2. Nutrition

In the present study, ultramarathon runners demonstrated significantly superior overall nutritional knowledge compared with American football players. Additionally, there was a significant positive association between nutritional knowledge and diet quality in AFs. However, dietary habits themselves were comparable in both studied groups.

There is limited research directly comparing nutritional knowledge and dietary patterns between endurance and strength-oriented athletes. Based on our findings, we cautiously speculate that the relatively low BMI observed in endurance athletes may reflect the specific physiological demands of their sport, where maintaining a lean phenotype is closely linked to performance. This may necessitate greater attention to energy balance, macronutrient distribution, and overall diet quality, potentially fostering higher nutrition-related awareness. In contrast, strength-focused athletes, such as football players, prioritize muscle mass and total body weight, and strict regulation of body fat or energy intake may be less critical for performance. Consequently, differences in sport-specific body composition requirements could underlie the observed variation in nutritional knowledge between these groups. Interestingly, while UMs demonstrated significantly higher nutritional knowledge compared to AFs, this advantage did not translate into statistically different dietary habit scores between the two groups. This discrepancy may be explained by differences in the athletes’ daily training environments. AFs often operate within highly structured team settings where nutrition is externally managed by coaching staff and sport dietitians, enabling them to maintain appropriate dietary behaviors without necessarily possessing deep theoretical knowledge. This observation is in line with previous findings in football players, suggesting that diet quality and access to professional nutritional support may play a role in training outcomes and overall athlete management [32]. Conversely, UMs are typically independent athletes whose dietary habits rely heavily on intrinsic knowledge. Furthermore, it is plausible that while UMs possess superior knowledge regarding specific peri-workout strategies (e.g., carbohydrate periodization or fluid management during ultra-endurance events), these sport-specific applications are not fully captured by general dietary habit questionnaires, resulting in similar overall habit scores between the cohorts.

Despite these sport-specific demands, existing evidence suggests that athletes in both endurance and team/strength disciplines often fall short of recommended dietary standards. Team-sport athletes frequently consume inadequate amounts of fruits, vegetables, whole grains, and dairy, with many classified as having “inadequate” diets [33], and have nutritional knowledge scores around 55–57% of the possible maximum with no clear association with body composition or energy intake [34]. Adolescent football players and professional soccer athletes similarly exhibit poor diet quality and insufficient carbohydrate intake [35]. Likewise, ultramarathon and marathon runners, despite high levels of physical activity, often show suboptimal intake of core food groups, inconsistent macronutrient distribution, and inadequate hydration and race-day fueling strategies [36,37]. These findings highlight a persistent need for targeted nutritional interventions across both endurance and strength-oriented athlete populations.

### 4.3. Sleep

In our study, football players exhibited significantly poorer sleep quality compared with UM, as reflected by higher global PSQI scores (Mean = 6.33 vs. 4.53). Deficits among AFs were particularly evident in sleep latency, daytime dysfunction, and subjective sleep quality, while differences in sleep disturbances, habitual sleep efficiency, and use of sleep medications were not significant. Self-reported sleep quality further supported this pattern, with 73.4% of runners reporting good sleep versus only 55.8% of AFs. These differences may also reflect psychological and behavioral characteristics associated with participation in different sport disciplines. Endurance sports such as ultramarathon running often require high levels of self-regulation, long-term health orientation, and structured lifestyle habits, which may be associated with more favorable sleep behaviors [38]. In contrast, team and contact sports such as football may attract athletes with a stronger focus on strength, competition, and physical dominance, potentially accompanied by different motivational profiles and lifestyle patterns that could influence sleep behaviors.

These findings are consistent with existing research indicating that team-sport and strength-oriented athletes often experience lower sleep quality. For example, team-sport athletes report disrupted sleep behaviors, including frequent nocturnal awakenings and thermal discomfort [39], and college American football players average only 6.2 h of sleep per night, below recommended levels [40]. PSQI scores in comparable football cohorts have ranged from 7 to 8, indicating generally poor sleep quality, similar to the elevated PSQI observed in our football sample (mean = 6.33) [41,42]. Conversely, endurance athletes often demonstrate relatively better sleep quality, although a 2021 study reports shorter sleep duration in runners compared to athletes in team sports [43].

Several factors may help explain the poorer sleep quality observed among football players in our study. Body composition characteristics common in football, particularly linemen, such as higher BMI, larger waist and neck circumferences, and greater body fat, increase the risk of sleep-disordered breathing, including obstructive sleep apnea, which is highly prevalent in this population and contributes to disrupted sleep [44,45]. In addition, the demands of team sports, including late training sessions, evening-oriented schedules, and competition timing, can delay bedtimes and reduce total sleep duration [39,46]. Psychological stressors, such as anxiety, depression, and general mental fatigue, have also been linked to sleep disturbances in contact sport athletes [47,48]. In contrast, endurance athletes typically maintain lower body mass and leaner physiques [49], and they often have greater control over their training schedules, which may support earlier bedtimes, more regular circadian rhythms, and overall better sleep quality [46]. Collectively, these physiological, behavioral, and environmental factors likely contribute to the sport-specific differences in sleep observed between football players and ultramarathon runners.

### 4.4. Sleep and Nutrition

Importantly, while initial analyses suggested potential correlations between DQI and certain sleep parameters within the AF cohort, these associations did not remain significant as independent predictors in the multivariable regression model. This loss of significance may be attributed to the dominant influence of stronger predictors, such as sport discipline and BMI, which could have masked the effects of dietary variables, particularly within the constraints of a relatively small sample size. Furthermore, the underlying relationship between diet and sleep may be non-linear; for instance, a threshold effect may exist wherein dietary quality impacts sleep only up to a baseline level of nutritional adequacy, beyond which it provides no further measurable benefit. Consequently, these specific dietary associations must be interpreted with caution. Future studies utilizing larger sample sizes and allowing for non-linear modeling are necessary to definitively elucidate the nuanced relationships between dietary habits and sleep architecture in athletic populations.

Our study found that among UM, correlations between dietary indices and PSQI components were generally weak and not statistically significant. In contrast, AF exhibited several significant associations: adherence to PHD was linked to shorter sleep latency, reduced daytime dysfunction, and better overall PSQI scores, while an unhealthy diet was associated with greater sleep disturbances and poorer global sleep quality. Additionally, BMI in AFas was positively correlated with sleep medication use, suggesting a link between body composition and sleep management. The stronger statistical relationships observed in our AF cohort may reflect their generally poorer baseline sleep and dietary habits compared with UMs, making these associations more apparent.

These associations may also reflect physiological and lifestyle differences between the studied groups. In athletes with higher body mass, sleep disturbances may be more prevalent due to increased risk of conditions such as obstructive apnea, which has been strongly associated with higher BMI and may contribute to sleep fragmentation and poorer overall sleep quality [50,51]. Interestingly, despite robust existing scientific evidence, our study found that higher BMI was associated with lower PSQI scores. This unexpected finding may reflect the specific characteristics of the studied cohort. In strength–power athletes, higher BMI often corresponds to greater lean body mass rather than excess adiposity, which may not be associated with typical risk factors for sleep-disordered breathing [52]. Alternatively, this observation may be influenced by the subjective nature of the PSQI questionnaire, which may not capture subclinical sleep disturbances such as undiagnosed sleep apnea [53]. It is also possible that athletes with higher BMI receive more structured medical support or recovery management within team settings, which could partially mitigate sleep-related impairments.

Furthermore, the intermittent, high-intensity, and contact-based nature of football training may lead to greater physiological stress, muscle damage, and inflammatory responses compared with the predominantly endurance-based training typical of UMs. Under such conditions, dietary quality, particularly anti-inflammatory versus highly processed dietary patterns, may be associated with sleep outcomes, although no independent effect was observed in the adjusted analyses [54]. In Australian football players, evening dietary patterns, particularly protein and sugar intake, and the timing of the evening meal relative to bedtime were associated with total sleep duration and efficiency [55]. Similarly, university athlete samples show modest but significant relationships between sleep quality and dietary behaviors, with poorer sleep linked to suboptimal nutrition, such as frequent fast-food consumption and meal skipping [56]. Dietary modifications and proper sleep hygiene represent universal strategies for health improvement. This is supported by research on sleep disorders such as bruxism, where daily habits and diet play a crucial role in alleviating symptoms [57]. This suggests that optimizing nutrition in athletes may yield benefits extending beyond post-exercise recovery to support physiological well-being.

However, contrary to our initial expectations and findings in general populations, we did not observe a significant relationship between overall dietary habits and sleep quality in the studied cohorts. Similar results have been reported in recent research on student athletes, where no association was found between Mediterranean diet adherence and PSQI scores [58]. One possible explanation is that athletes may already meet a nutritional baseline sufficient to support their physiological demands, resulting in limited variability in diet quality. In highly trained individuals, compensatory mechanisms may further buffer the impact of subtle dietary differences on sleep. This suggests that athletes may have a higher tolerance to dietary variation before sleep is affected. Rather than diminishing the role of nutrition, these findings highlight the multifactorial nature of sleep in athletic populations and point to a potential ceiling effect, where general dietary quality alone is insufficient to offset other influential factors such as training load, competition-related stress, or suboptimal sleep hygiene. Consequently, a holistic approach to recovery is warranted, integrating nutritional strategies with targeted behavioral interventions to effectively improve sleep quality.

Future research should explore the effectiveness of sport-specific nutritional education and individualized dietary counseling in improving diet quality and performance outcomes. Longitudinal studies are needed to examine how changes in body composition, dietary habits, and sleep quality interact across different phases of training and competition. Finally, organizational support, including access to dietitians, sleep clinics, and structured recovery resources, is essential to implement these practices effectively and promote sustainable athlete health.

### 4.5. Limitations

This study has several limitations that should be acknowledged. The relatively small overall sample and the substantial difference in group sizes may limit statistical power and the generalizability of the findings. It should also be noted that the use of random undersampling to balance group sizes led to the exclusion of a large proportion of the UM group (n = 140), which may have reduced statistical power and affected the representativeness of the analyzed sample. Data were collected via an online survey, which, despite careful efforts to provide clear instructions, may have led to misinterpretation of questions by some participants. No a priori power calculation was performed due to the exploratory nature of the study, which may limit the statistical inference of the findings. Furthermore, the cohort consisted entirely of male athletes, and critical metrics such as BMI and sleep quality (evaluated via the PSQI questionnaire) were derived from subjective, declarative responses rather than objective methods. Additionally, the cross-sectional design of the study prevents the establishment of direct cause-and-effect relationships, and the absence of control for confounding factors (e.g., collision versus non-collision sports, body composition, training structure) limits the interpretability of the findings. Consequently, the results should be interpreted with caution and should not be generalized to all UM or AF. Additionally, BMI was self-reported and does not reflect body composition, which limits the interpretation of findings related to this variable. Therefore, differences observed between groups should be interpreted cautiously and cannot substitute for direct body composition measurements. Given the sample size and number of predictors, the regression model may be susceptible to overfitting, and the identified associations should be considered exploratory rather than definitive.

Overall, the interpretation has been restricted to findings that remained significant after the FDR correction and multivariable modeling to avoid overinterpretation.

## 5. Conclusions

The present study demonstrated clear sport-specific differences in BMI, nutritional knowledge, dietary habits, and sleep quality between UMs and AFs. UMs demonstrated more favorable BMI profiles, higher nutritional knowledge, and better overall sleep quality. In contrast, AFs more frequently presented with higher BMI and lower sleep quality. However, a positive association observed in the AF group between DK and dietary habits suggests a potential area for targeted recommendations.

These findings highlight the importance of considering sport-specific physiological and behavioral characteristics when designing strategies to optimize nutrition, sleep, and recovery in athletes. However, dietary variables were not independently associated with sleep quality in the multivariable model and should not be overinterpreted.

## Figures and Tables

**Table 1:** Characteristics of the baseline group.

Variables	Runners, *n* = 188 [IQR] (%)	Football Players, *n* = 43 [IQR] (%)
Gender		
Male	188 (100.0)	43 (100.0)
Place of residence		
Rural area	34 (18.1)	7 (16.3)
City < 50,000 *	41 (21.8)	8 (18.6)
City 50,000–100,000 *	17 (9.0)	0 (0.0)
City 100,000+ *	96 (51.1)	28 (65.1)
Body Mass Index		
Mean	24.1 [22.6–25.4]	29.1 [25.9–31.7]
Underweight	2 (1.1)	0 (0.0)
Normal weight	118 (62.8)	7 (16.3)
Overweight	68 (36.2)	36 (83.7)
Nutritional knowledge mark		
Insufficient	17 (9.0)	0 (0.0)
Sufficient	125 (66.5)	43 (100.0)
Good	46 (24.5)	0 (0.0)
Sleep quality		
Good	138 (73.4)	19 (55.8)
Poor	50 (26.6)	24 (44.2)

Note: *n* is the number of observations; * number of inhabitants.

**Table 2:** Categorical characteristics of the balanced groups (*n* = 86).

Variable	Category	AF	UM	*p*-Value (Fisher)
BMI	Normal	7 (16.3%)	30 (69.8%)	<0.001
	Overweight	36 (83.7%)	13 (30.2%)	<0.001
PHD	Low	34 (79.1%)	29 (67.4%)	0.330
	Medium	9 (20.9%)	14 (32.6%)	0.330
NHD	Low	38 (88.4%)	43 (100%)	0.055
	Medium	5 (11.6%)	0 (0%)	0.055
DK	Good	0 (0%)	12 (27.9%)	<0.001
	Sufficient	43 (100%)	28 (65.1%)	<0.001
	Insufficient	0 (0%)	3 (7%)	<0.001
Sleep quality	Good	19 (44.2%)	29 (67.4%)	0.050
	Poor	24 (55.8%)	14 (32.6%)	0.050

Note: AF—American football players, UM—ultramarathon runners, BMI—Body Mass Index, PHD—Pro-Healthy Diet Index, NHD—Non-Healthy Diet Index, DK—Dietary Knowledge.

**Table 3:** Comparison of all analyzed continuous variables (*n* = 86).

Variable	AF [IQR]	UM [IQR]	Corrected *p*-Value (FDR)	Effect Size (r)
BMI	28.72 [5.72]	24.57 [2.78]	<0.001	0.632
PHD	23.8 [6.25]	25.9 [13.45]	0.491	0.082
NHD	15.8 [16.9]	13 [8.55]	0.130	0.192
DQI	7.6 [17.2]	12.1 [18.55]	0.040	0.255
DK	11 [2]	15 [5.5]	<0.001	0.499
PSQI Total Score	6 [5]	5 [3]	0.026	0.280
C1	1 [0]	1 [0]	0.048	0.244
C2	1 [2]	1 [1]	0.026	0.275
C3	0 [0.5]	0 [1]	0.491	0.083
C4	0 [0]	0 [1]	0.194	0.164
C5	1 [0]	1 [0]	1.000	0.000
C6	0 [0]	0 [0]	0.484	0.099
C7	2 [1]	1 [2]	0.003	0.366

Note: AF—American football players, UM—ultramarathon runners, FDR—False Discovery Rate, BMI—Body Mass Index, PHD—Pro-Healthy Diet Index, NHD—Non-Healthy Diet Index, DQI—Diet Quality Index, DK—Dietary Knowledge, PSQI—Pittsburgh Sleep Quality Index, C1—subjective sleep quality, C2—sleep latency, C3—sleep duration, C4—habitual sleep efficiency, C5—sleep disturbances, C6—use of sleep medication, C7—daytime dysfunction.

**Table 4:** Integrated multiple regression model for the global sleep quality score.

Term	Estimator	95% CI	*p*-Value
Intercept	13.912	8.726 to 19.097	<0.001
Athletic group (UM)	−2.498	−3.976 to −1.021	0.001
BMI	−0.229	−0.400 to −0.058	0.008
DQI	−0.018	−0.065 to 0.029	0.450
DK	−0.071	−0.242 to 0.100	0.414

Note: UM—ultramarathon runners, BMI—Body Mass Index, DQI—Diet Quality Index, DK—Dietary Knowledge.

**Table 5:** Targeted Spearman correlations between BMI and key PSQI components by sport discipline (*n* = 43 per group).

Group	PSQI Component	Spearman’s Rho	Corrected *p*-Value (FDR)
AF	Global PSQI score	−0.350	0.021
	C2	−0.449	0.008
	C7	−0.392	0.014
UM	Global PSQI score	−0.084	0.768
	C2	−0.226	0.433
	C7	0.046	0.768

Note: AF—American football players; BMI—Body Mass Index; UM—ultramarathon runners, PSQI—Pittsburgh Sleep Quality Index, FDR—False Discovery Rate, C2—sleep latency, C7—daytime dysfunction.

**Table 6:** Targeted Spearman correlations between DQI and key PSQI components by sport discipline (*n* = 43 per group).

Group	PSQI Component	Spearman’s Rho	Corrected *p*-Value (FDR)
AF	Global PSQI score	0.135	0.580
	C2	0.055	0.724
	C7	0.273	0.228
UM	Global PSQI score	−0.092	0.560
	C2	0.091	0.560
	C7	−0.225	0.441

Note: AF—American football players; UM—ultramarathon runners, PSQI—Pittsburgh Sleep Quality Index, FDR—False Discovery Rate, C2—sleep latency, C7—daytime dysfunction.

**Table 7:** Spearman correlations between nutritional knowledge and DQI by sport discipline.

Group	Variables	Spearman’s Rho	*p*-Value
AF	Nutritional Knowledge vs. DQI	0.388	0.010
UM	Nutritional Knowledge vs. DQI	−0.149	0.341

Note: AF—American football players; UM—ultramarathon runners, DQI—Diet Quality Index.

## Data Availability

The datasets used and analyzed during the current study are available from the corresponding author upon reasonable request. The data are not publicly available due to the inclusion of information that could compromise the privacy of the research participants in accordance with the decision of the Ethics Committee of the Wroclaw Medical University.

## Abbreviations

The following abbreviations are used in this manuscript:

AF	American football players
ALMI	Appendicular lean mass index
BMI	Body Mass Index
C1	Subjective sleep quality
C2	Sleep latency
C3	Sleep duration
C4	Habitual sleep efficiency
C5	Sleep disturbances
C6	Use of sleep medication
C7	Daytime disfunction
DK	Dietary Knowledge
DQI	Diet Quality Index
FDR	False Discovery Rate
IQR	Interquartile Ranges
KNOWL	Knowledge about nutrition
MEDS	Use of sleep medication
NHD	Non-Healthy Diet Index
NUTRSC	Nutrition score
PHD	Pro-Healthy Diet Index
PSQI	Pittsburgh Sleep Quality Index
TOTAL	Total score
UM	Ultramarathon Runners
